# Skull Base Inverted Papilloma: A Comprehensive Review

**DOI:** 10.5402/2012/175903

**Published:** 2012-12-31

**Authors:** Shafik N. Wassef, Pete S. Batra, Samuel Barnett

**Affiliations:** ^1^Department of Neurosurgery, University of Iowa Hospitals and Clinics, Iowa City, IA 52242, USA; ^2^McConnell Brain Imaging Center, Montreal Neurological Institute and Hospital, McGill University, Montreal, QC, Canada H3A 2B4; ^3^Department of Neurology and Neurosurgery, McGill University, Montreal, QC, Canada H3A 2B4; ^4^Department of Otolaryngology—Head and Neck Surgery, UT Southwestern Medical Center, Dallas, TX 75390, USA; ^5^Department of Neurosurgery, UT Southwestern Medical Center, Dallas, TX 75390, USA

## Abstract

Skull base inverted papilloma (IP) is an unusual entity for many neurosurgeons. IP is renowned for its high rate of recurrence, its ability to cause local destruction, and its association with malignancy. This paper is a comprehensive review of the reports, studies, and reviews published in the current biomedical literature from 1947 to September 2010 and synthesize this information to focus on its potential invasion to the base of the skull and possible intradural extension. The objective is to familiarize the clinician with the different aspects of this unusual disease. The role of modern diagnostic tools in medical imaging in order to assess clearly the limits of the tumors and to enhance the efficiency and the safety in the choice of a surgical approach is pointed out. The treatment guidelines for IP have undergone a complex evolution that continues today. Radical excision of the tumour is technically difficult and often incomplete. Successful management of IP requires resection of the affected mucosa which could be achieved with open surgery, endoscopic, or combined approach. Radio and chemotherapy were used for certain indications. More optimally research would be a multicenter randomized trials with large size cohorts.

## 1. Background

Inverted papillomas generate considerable interest because they are locally aggressive, have a propensity to recur, and are associated with malignancy [1, 2]. Recurrent disease and metachronous carcinoma can develop after a prolonged period of time [2]. Skull base inverted papilloma is a benign sinonasal neoplastic proliferation. Papilloma per se lacks the essential criteria for malignancy, such as metastasis. It has the propensity for invasion into adjacent structures, such as the orbit and CNS, even in the absence of malignancy. Intracranial involvement of inverted papilloma is unusual and is usually seen in recurrent cases [3]. Recognition of the propensity for recurrence and the association with malignancy has led to the evolution of treatment. Many aspects of sinonasal inverted papillomas are still controversial [4] and active fields of research. This could be more challenging in a case of intracranial with intra- or extradural extension.

## 2. Definition

The term *papilloma* means neoplasia with epithelial growth. The US National Cancer Institute's [5] has defined inverted papilloma as a type of tumor in which surface epithelial cells grow downward into the underlying supportive tissue. The term inverted is derived from the characteristic proliferation of metaplastic surface epithelium (respiratory, transitional, or squamous type) by inversion into the underlying stroma, resulting in endophytic rather than exophytic growth [6]. It may occur in the nose and/or sinuses or in the urinary system. When it occurs in the nose or sinuses, it may cause symptoms similar to those caused by sinusitis, such as nasal congestion.

## 3. History

In 1600s, C. Victor Schneider demonstrated that nasal mucosa produces catarrh and not CSF and identified its origin from the ectoderm. The first report of this type of tumour in the nasal cavity was made by Ward in 1854 [7]. Ringertz et al. [8–10] in 1938 was the first to identify endophytic growth pattern of IPs with its characteristic tendency to invert into the underlying connective tissue stroma, which differs from other types of papillomas, and called it “inverting papilloma.” Kramer et al. classified IPs as true nasal neoplasms and described them as genuine papilloma of the nasal cavity, distinguishing them from inflammatory nasal polyps [11].

## 4. Histopathology

### 4.1. Relevant Histology

The lining of the nasal cavity and paranasal sinuses is unique in the upper aerodigestive tract in that it is ectodermal in origin. It is formed of ciliated, pseudostratified columnar epithelium, the Schneiderian membrane, with a thin submucosa containing seromucous glands. The Schneiderian membrane is of ectodermal origin from the nasal placode [12]. The submucosa is very vascular in the nasal cavity, but in the sinuses the lining is thinner and less vascular, with a fibrous layer adjacent to the periosteum. The roof of the nasal cavity is lined with olfactory neuroepithelium. Tumors peculiar to the region therefore include the Schneiderian inverted papilloma and olfactory neuroblastoma. The commonest neoplasms, however, are those arising from metaplastic epithelium—squamous cell carcinoma (SCC)—and from the mucoserous epithelium-adenocarcinomas and other tumors. Non-epithelial tumors are similar to those found elsewhere in the head and neck [13].

Inverted papilloma arises from the outlining Schneiderian respiratory membrane [14]. The behavior of the invasion into the underlying stroma was attributed to its origin from the Schneiderian membrane, as there may be some difference in the underlying stroma which permits inversion of the papilloma [12, 15–19]. In the English-speaking area, it is also called “inverted Schneiderian papilloma,” indicating its origin from the Schneiderian membrane. *Schneiderian papillomas* most often arise from the ectodermally derived mucosa of the nasal cavity and sinuses (Schneiderian epithelium).

### 4.2. Classification

The histomorphologically based classification formulated by Hyams (1971) [21, 22] divided these polyps (papillomas of the sinonasal tract) due to their pattern of growth into three histological categories and their malignant counterparts [21, 23], and they are classified as follows (see Table 1):

- *fungiform (everted) papillomas (septal papilloma) (50%):* arise from the nasal septum and have an exophytic growth pattern,
- *oncocytic Schneiderian papillomas*: formerly known as *“cylindrical cell papillomas*,*”* arise from the lateral sinus wall or paranasal sinuses, and lined with a distinctive stratified lining of bright pink granular columnar cells with microcysts, and more often mixed with typical inverted papilloma, rather than presenting in its pure form [24–34],
- *inverted papillomas:* arise from the lateral nasal or sinus wall or paranasal sinuses and show an endophytic or mixed exophytic/endophytic growth pattern [35].

These tumors all are biologically similar [13]. The continued use of the term inverted papilloma (endophytic pattern) as a specific subset of Schneiderian papillomas is recommended, as all serious complications, including progression to local invasion, copresentation with carcinoma, and development of carcinoma, were associated with these lesions [32, 36–38].

The World Health Organization (WHO) classification basically followed this classification [39]. However, it should be noted that some authors regard the three types of nasal papilloma as three completely distinct entities [40], while others confirm that there are hybrid lesions combining features of IP and cylindrical cell papilloma in just one lesion [21, 24, 41]. 

### 4.3. Sites of Origin and Involvement

IP can be found in the lateral wall of the nose, nasal septum [42–44], the ethmoidal sinus [19, 45], the maxillary sinus, the sphenoidal sinus [46–55], the sphenoethmoidal recess [56], and the frontal sinus [57–62]. IP most commonly arises from the lateral nasal wall [63–66]. Incidence of isolated sphenoid IP is exceedingly low [46–55, 67, 68]. Bilateral IP and multifocal involvement have been reported in the literature but still are an uncommon finding [69–80]. Common sites of intracranial spread include the cribriform plate, fovea ethmoidalis, and orbits [3, 81]. 

### 4.4. Pathology: Macroscopically

Grossly, inverted papilloma looks like a polyp with a narrow or broad-based stalk, but is usually firmer with significant bulk and has more of a granular mulberry type appearance. They are a variety of colors from red to pale pink. IPs have an irregular, friable appearance. They are usually more vascular than the average polyp and bleed easily. IP must be distinguished from squamous papillomas, which consist of an arborizing fibrovascular core with a bland squamous epithelial lining and from sinonasal polyps, which represent localized outpouchings of the sinonasal mucosa due to edema [23, 24, 82, 83].

### 4.5. Pathology: Microscopic

Microscopic features are that of digitiform proliferation of squamous epithelium with extensive invasion of the hyperplastic epithelium into the underlying stroma. The tumor has crypts which are subepithelial and maintain connection to the surface epithelium at all times; a finding which lead to the name inverted papilloma. Microcysts containing mucus are often trapped within the neoplastic epithelium. The lining consists of multilayered nonkeratinized squamous epithelium with scattered mucous cells and microcysts filled with neutrophils. The covering epithelium can be squamous, respiratory, or transitional cell epithelium or a combination of all three [66]. The cells show minimal nuclear atypia with the typical basilar layer mitosis. The stroma usually has acute and chronic inflammatory changes with areas of fibrosis and edema. The stroma is almost lacking in eosinophils which would be prevalent in an allergic polyp [84, 85]. Inflammatory cells were identified as a significant cell population in IP, whereas it was less commonly encountered in other forms of SP [86].

On the bases of electron density, cells from inverted papilloma specimens are characterizable as light, intermediate, and dark cells. This variation reflects variations in the amount of granular endoplasmic reticulum and non-membrane-bound polysomes. Cytological characteristics include filament formation, filament concentration into bundles, and changes in nuclear structure leading to pyknosis [87].

### 4.6. Pathology: Neoplastic Change

Metaplastic change might play an important role in the malignant transformation of IP. Evidence suggests that IP originates in submucosal glands, specifically exocrine glands in the sinonasal region. In these glands, metaplastic change was commonly seen and occasionally revealed malignant features such as SCC in the sinonasal cavity [88]. Features associated with recurrence or malignant transformation include bone invasion, high grade dysplasia, increased mitotic activity, epithelial hyperplasia, and epithelial overgrowth of stroma. Favorable histologic features include the presence of inflammatory polyps and high eosinophil counts [89–91]. These lesions have definite rates of conversion to or harboring of invasive SCC that requires adequate evaluation for diagnosis and treatment selection [92]. Association with adenocarcinoma has also been reported [93].

## 5. Skull Base Invasion

### 5.1. Local Bone Destruction

As many as 75% of patients with IP have evidence of various degrees of bone destruction. These may include thinning, remodeling, erosion, and (less commonly) sclerotic bony changes. Benign sinonasal masses and slow-growing neoplasms tend to remodel the nasal vault and facial bones, and this is particularly true of nasal polyps and inverted papillomas. However, when such benign masses press against the floor of the anterior cranial fossa and the walls of the sphenoid sinuses, simulated aggressive bone destruction rather than bone remodeling usually occurs [94]. The presence of bone destruction alone does not indicate differentiation into malignancy from the IP. In fact, about 90% of the time no carcinoma is present with IP [94]. On histopathologic analysis, the bony surface underlying the IP was irregular with multiple bony crevices. This irregularity of the bony surface may hinder complete tumor removal because microscopic nests of mucosa can be hidden within the bony crevices. CT scanning is more precise than conventional radiography for identifying the areas of bony erosion. Radiographic osteitic bony changes were seen at site of tumor attachment. Intraoperative removal of the bony surface at the site of tumor attachment may ensure a more complete removal [95].

This type of bone destruction implies to the radiologist that a carcinoma may also be present [96], and this information could dissuade a surgeon from operating with an attempt at cure. The pressure of the growing IP causes also erosion of bone [97]. In the event of direct invasion and osteolysis, the presence of associated malignancy has to be considered [94, 97–100]. IPs that invade the orbit have a high incidence of malignancy [58, 100].

### 5.2. Intracranial Extension

Benign and malignant IP can displace neighbouring structures, causing extension into the intracranial cavity with or without dural involvement [101]. Intracranial extension and dural penetration is rare and often associated with recurrent disease [102] that has degenerated into SCC [5] or less often transitional cell carcinoma [103]. The prognosis depends on the type of dural involvement, with intradural extension carrying a poorer prognosis [104].

 Extension to intracranial temporal fossa and middle ear has been reported in few cases in the literature [105]. Massive inverting papilloma with intracranial extension can cause marked frontal lobe compression [106].

Most tumors extended into the nasal cavity or ethmoid sinus combined frequently with erosion of the lateral wall or intersinus septum. Simultaneous attachment to multiple walls including both lateral wall and floor attachment was noted [68]. The complex nature of the anatomy of this region allows tumors from the sinuses to become involved early with the vital structures of the head and neck. In the past this was enough to deter most surgeons from even attempting to resect these malignancies and this in turn accounted in part for the poor prognosis in this group of diseases. Tumor may spread by direct invasion of bone and cartilage to involve related structures, but the walls of the nasal cavity and paranasal sinuses also contain numerous foraminae and fissures transmitting important neurovascular bundles [13]. Bony changes on CT imaging of inverted papilloma (IP) are useful for predicting tumor origin and recurrence sites [68].

Intracranial extradural inverted papilloma can be effectively controlled with craniofacial resection. Intracranial intradural involvement of inverted papilloma has a poor prognosis regardless of treatment. Aggressive treatment of intranasal inverted papilloma may be the most important factor in preventing intracranial presentation [102, 107].

## 6. Etiology and Pathogenesis

The etiology of inverted papillomas is still unclear. There have been many causes suggested such as allergy, chronic sinusitis [108], viral infections, and Inflammation [86, 109]. Allergy, however, is unlikely since most of the patients do not have an allergic history and the polyposis associated with allergic rhinitis is usually bilateral. The presence of sinusitis is thought more to be related to the obstructive nature of the disease and not the cause. The signs of chronic infection of the sinuses are much too common to be implicated in the rare case of inverted papilloma. 

Histological analysis suggests that IP tumorigenesis may occur through a stepwise series of discrete events graded according to a four-stage histological grading system (stages I and II, benign IP; stage III, dysplastic IP; stage IV, carcinoma arising from IP). Viral infection and genetic insults may be required to progress [110–112]. Inducing or promoting agents have been suggested in the pathogenesis of this disease. These include human papilloma virus, alterations in tumor suppressor gene p53 [113], and chronic inflammation [86, 114, 115]. Occupational exposure [116, 117] to different smokes, dusts, and aerosols noxious agents may play a possible role in the pathogenesis of IP [108, 118].

Viruses have been implicated due to the well described finding in recurrent respiratory papillomatosis. There is little consensus regarding the incidence or role of Epstein-Barr virus (EBV) in IP [119, 120] which is further questioned by other studies [121, 122]. Human papilloma virus (HPV) infection is responsible for 15 to 35 percent of head and neck cancers worldwide [123–130]. HPV has been etiologically associated with a subset of sinonasal papillomas and squamous carcinoma, and those with benign and malignant clinical course are separable on basis of HPV type [131–134]. HPV infection might be one of the possible causative factors in the pathogenesis of IP [110, 135, 136] but is not essential for the induction of IP [137, 138]. In terms of aetiology there is some evidence that the presence of HPV in IP could be involved in the malignant transformation [111, 139–145] and that HPV subtyping enables a prognosis of the probability of recurrence [146]. Significant association has been identified between the presence of human papilloma virus DNA in inverted papilloma and recurrence after surgical excision [144, 147, 148]. Some authors argued against the role of HPV in the pathogenesis of exophytic papilloma while others denied its association with developing malignancy and recurrence [121, 149–151]. HPV serotypes 6 and 11 are associated with the fungiform papilloma, and to a lesser degree, with inverted papillomas [37, 120, 152–156]. Patients with proof of HPV types 6 or 11 have a lower rate of recurrence than patients with HPV types 16 or 18 [147, 147, 157–159]. HPV 16 and HPV 18 were found to be related to the malignant transformation of IP and to the pathogenesis of SCC originated in the nasal cavities and paranasal sinuses [114, 143, 159–164]. HPV 57 was isolated from inverted papilloma of the nasal cavity and paranasal sinuses [165] and is possibly associated with premalignant and malignant IP [141, 166]. 

The observation that IP tends to recur after incomplete surgical removal supports investigations suggested that IP is a true neoplasm arising from a single progenitor cell and that recurrence represents growth of the residual clone [167]. Although IPs are monoclonal proliferations, they do not fit the profile of a prototypic precursor lesion. Unlike squamous epithelial dysplasia, IPs do not routinely harbor several of the key genetic alterations that are associated with malignant transformation of the upper respiratory tract [167, 168]. Flow cytometric analysis of DNA content from paraffin-embedded material is a diagnostic and prognostic method in clinical pathology and investigative oncology. DNA histogram is a good indicator for biological activity of head and neck cancers. The increased S + G2M% and polyploid% may indicate carcinogenic process [169]. Increased epithelial cell proliferation, but not apoptosis and apoptosis inhibition, seems to be involved in the development of IP [170, 171]. An increased epidermal growth factor receptor (EGFR) and Tumor growth factor- (TGF-) alpha expression is associated with early events in IP carcinogenesis [139, 140, 172–174]. 

CIP/KIP family proteins entitled p21(WAF1/CIP1) and p27(KIP1) have key positions in cell cycle regulation leading to an arrest of cell proliferation. They are supposed to enable a repair process of DNA damage. In several human tumors, a loss of these proteins is associated with poor clinical outcome [175]. IP contains a significantly higher cell population with proliferative activity by comparison with normal sinonasal and inflammatory polyp epithelia, showing a significant correlation between Topoisomerase II-alpha (topoII-alpha) and Ki67 expression, and indicating that topoII-alpha could be a independent prognostic factor for a putative malignant transformation [176, 177]. Immunohistochemical staining of inverted sinonasal papillomas for p53 [115, 175, 178–187] and Ki67 can give useful information concerning the existence of synchronous carcinoma [173, 188] and, in case of high Ki67, a hint toward possible recurrence [136, 177, 189, 190, 190–193]. A study demonstrated ectopic production of beta-human chorionic gonadotropin in cases of inverted papilloma of the nose [194]. Immunohistochemical analysis of the cytokeratin profile revealed increased expression of cytokeratin 5, typical for basal cells, and cytokeratin 13, typical for squamous epithelial cells. This suggests that IP is derived from a cytokeratin 5-immunoreactive cell of the basal layer of the mucosa [195, 196]. Neuraminidase pretreatment (NA-PNA) staining may be helpful for an early detection of IP. Strong NA-PNA staining in IP may predict malignant transformation [197]. As in other papillary epithelial neoplasms, the typical benign IP features diffuse membranous CD44s expression. In cases of IP developing an invasive SCC, CD44s expression in the SCC component is frequently lost [198, 199]. Loss of p27 expression or its reduced level correlates with increased cell proliferation in sinonasal tumours [136, 191]. p27 expression may be associated with keratinization in SCCs of the paranasal sinus and therefore may be a useful marker for the dysregulation of cell kinetics in IP [200–203]. Expression of p21 (waf1/cip1) is associated with terminal differentiation in surface cells in inverted papillomas and cylindrical cell papillomas [136, 203, 204]. Serum SCC antigen may be a useful biologic marker in patients with sinonasal IP [205–207]. Proliferating cell nuclear antigen labeling index (PCNA-LI) can be used as a strong independent indicator to judge tumor aggressive behavior, proliferative activity, development of local or regional metastases, and prognosis in nasal inverted papilloma [208, 209].

## 7. Epidemiology

Inverting papilloma is a relatively uncommon neoplasm of the nasal cavity, constituting 0.5% to 4% of all primary nasal tumors [6, 210–216] with an incidence of 0.6–1.5 cases per 100,000 per year [217–219]. Although the age range for occurrence is 6–102 years [220, 221], SPs are rare in children [222] and young adults [79, 223, 224]. Patients with carcinoma in IP are older than those IP patients without carcinoma [16, 36, 225] and are predominantly male [225, 226]. The peak incidence is in the fifth and sixth decades of life [211, 227, 228], but it has been reported in all age groups [6, 229, 230]. Clearly, men are affected more often than women [3, 228, 231, 232] with a 3 to 1 male to female predominance ratio [66, 230]. Caucasians are more commonly affected—white persons are most at risk, compared with persons of other races. Finally, about 5 to 15 percent of patients are associated with squamous cell carcinoma either subsequently or concurrently [2, 6, 79, 211, 232–236]. This emphasizes the importance of wide excision and long-term followup for affected patients. 

## 8. Clinical Presentation

### 8.1. Symptoms

Since the nasal cavity and sinuses are air-filled cavities, tumors must reach a significant size before they cause symptoms. Symptoms usually do not develop unless the involved sinus cavity is obstructed or the tumor has extended beyond the confines of the sinus, producing symptoms secondary to invasion of adjacent structures (see Tables 2 and 3). Early neoplasms tend to be asymptomatic until they invade adjacent structures, at which time symptoms may mimic benign diseases [237, 238]. Early lesions are incidentally diagnosed on radiological studies taken for other disease, for example, inflammatory or trauma. When symptoms do occur, they are nonspecific, mimicking a host of far more common benign problems such as sinusitis, headache, and toothache, and are disregarded by both the patients and the doctors caring for them. The time between the onset of symptoms and a diagnosis of cancer averages from 6 to 12 months [13, 239, 240]. The limited anatomic access of the paranasal sinuses makes early diagnosis difficult. For all of these reasons, most patients have advanced disease by the time of diagnosis [241–245].

Inverted papilloma often manifest as unilateral polyps [235]. The most often noted revealing sign is unilateral nasal obstruction [45, 231, 246–251], but they can also cause bleeding [252], or sinusitis [109, 253]. When symptoms of persistent nasal discharge and epistaxis [45] occur in patients over age 40, clinicians may want to include paranasal sinus cancer in the differential diagnosis [254–256]. Epistaxis, obstruction, or sinusitis which is unilateral should raise the index of suspicion for the possibility of a neoplastic process [13]. For inverted papilloma originating from the sphenoid sinus, the common symptoms were headache and nasal obstruction [68]. The most common symptoms in patients with malignant transformation include facial or dental pain, nasal obstruction, and epistaxis [243, 244, 257]. Less common symptoms include cranial neuropathy, chronic sinusitis, facial edema, vision loss, headache, rhinorrhea, and hyposmia [243, 244]. A classic triad of facial asymmetry, palpable/visible tumor in the oral cavity, and visible intranasal tumor occurs in 40 to 60 percent of patients with advanced disease [258]. 

As disease progresses, symptoms and signs depend upon the involved site. The bony structures between the nasal cavity, sinuses, orbits, and cranial vaults are thin and offer little resistance to cancer spread (see Table 4). In the ethmoid sinus, locally advanced lesions may extend into the anterior cranial fossa via the cribriform plate or into the orbit through the lamina papyracea [259]. This may result in anosmia or displacement (typically upward and/or outward) of the globe. Orbital involvement is suggested by proptosis [259], medial canthal mass, periorbital swelling [260] and pain, or lacrimal drainage obstruction [100, 103, 261, 262]. Affected patients may complain of diplopia [260], blurred vision, inability to wear eyeglasses, epiphora, paresthesias in the distribution of the trigeminal nerve, or trismus if the pterygoid musculature is invaded [263, 264]. Patients may also experience retroorbital pain and visual loss or complain of facial numbness. In the sphenoid sinus, disease may directly extend through the lateral bony wall into the cavernous sinus. It may also invade the middle cranial fossa directly or via the infraorbital nerve. Direct extension through the eustachian tube may lead to the development of IP involving the middle ear and temporal bone [265]. Inferior extension into the oral cavity may also cause painful loose teeth [100]. They may have poorly fitting dentures or be aware of a mass in the oral cavity or have noticed swelling of the cheek, nose, or around the eye [13]. Meningitis has also been reported as a presenting symptom [266].

### 8.2. Clinical Examination

Physical examination may reveal the presence of a nasal mass or polyposis [267]. A mass may be present in the cheek or the medial canthus or be found as broadening of the nasal dorsum. Maxillary lesions may present in the oral cavity as a mass on the palate, the upper alveolus, or upper gingivobuccal sulcus or with malocclusion or loose teeth [268]. There may be impaired ocular mobility, either from direct orbital invasion or as a result of an abducent nerve palsy with sphenoid sinus involvement. Anesthesia or hypesthesia of the infraorbital, sphenopalatine, or greater palatine nerves may be present. Anosmia is infrequently a presenting complaint, but a common physical finding [240]. Trismus is a sign of advanced local disease. Facial swelling and proptosis (bulging of the eyes) may accompany lesions that have expanded considerably [99, 263, 264]. A neck mass is an uncommon finding on presentation [13]. 

The best means of examination is nasal endoscopy, as most inverted papillomas can be found during a physical examination of the nasal cavity. Endoscopic examination is necessary to assess local disease extent and to provide biopsy material. The macroscopic appearance has been described as that of a tumour with a mulberry-like uneven surface and a reddish grey-livid colour and may bleed when touched, but other colours can be encountered as well. The septum may be bowed by the mass. Nasal endoscopy may be a useful noninvasive exam to be used in the *in vivo* diagnosis and for the differential diagnosis between inverted Schneiderian papilloma, inflammatory polyps, and nasosinusal SCC and may enable better preoperative planning, even when the examiner is inexperienced [269, 270].

## 9. Work-Up Investigation

### 9.1. Laboratory

As with other head and neck cancers, liver enzymes are usually obtained to assess for distant disease in addition to a chest radiograph or CT scan to evaluate for pulmonary metastasis. In the case of a nasal cavity or paranasal sinus mass or erosion, an antineutrophil cytoplasmic antibody (ANCA) test for possible Wegener granulomatosis should be considered. This condition often mimics a neoplasm. Preoperative laboratory studies include a complete blood cell count, electrolyte evaluation, and bleeding and coagulation parameters. Masses located around the sella turcica merit serum endocrine studies.

### 9.2. Biopsy

Cytologic examination is a useful initial approach in the diagnosis of IP, and in the differential diagnosis of other tumors that occur in the same sites [271, 272]. Biopsy is necessary to make a definitive diagnosis [79]. Biopsy of lesions involving the maxillary sinus can usually be obtained in the outpatient clinic. Most lesions are accessible transnasally and endoscopes allow clear visualization to obtain adequate biopsies and thoroughly examine the nasal cavity. Where tumor is confined to the sinus, a biopsy should be obtained by direct access to the sinus. Biopsy can be performed intranasally or through the gingivo-buccal sulcus if the tumor extends through the anterior maxilla. Tumors of the maxillary antrum can be approached with a transnasal biopsy through the medial wall of the sinus. Ethmoid sinus lesions are biopsied through an endoscopic or transnasal approach in the operating room as are frontal sinus malignancies, via the frontal recess. Frontal sinus trephination is rarely necessary. Lesions presenting to the oral cavity can be biopsied transorally.

Surgical approaches may be necessary, although open biopsies should be avoided if possible to minimize the risk of contaminating otherwise noninvolved tissue planes. The maxilla can be approached through an anterior antrostomy through an upper gingivobuccal sublabial incision (Caldwell-Luc procedure) [273]. The ethmoids can be biopsied using endoscopic ethmoidectomy or an external ethmoidectomy through a Lynch incision. The sphenoid sinus can be approached endoscopically or transseptally. The frontal sinus can be approached through its floor [13].

### 9.3. Imaging

Imaging of the sinuses is essential before a treatment plan can be proposed to provide additional information for staging and treatment planning. The true extent of locally aggressive IPs cannot be accurately assessed on clinical examination because of their location within the bony craniofacial skeleton. There is currently not enough evidence to suggest one sole modality as providing optimum imaging for IP. The use of plain radiographic films and tomograms has given way to computed tomography (CT) and magnetic resonance imaging (MRI), which offer far more detailed information about the size and behavior of tumors [13, 274, 275]. CT and MRI are both typically performed for evaluation of sinus tumours, and IP should be included within this pathological group. CT and MRI are the techniques of choice for pretreatment staging in inverted papilloma (IP) [276]. CT and MRI imaging modalities are complementary in evaluating disease extent [231] and in distinguishing tumor from infection, retained secretions, and granulation or scar tissue [255]. Due to the nature of tumor histology occurring in the paranasal sinuses, CT offers superior bony definition and MRI gives superior soft tissue delineation [277, 278]. The cohort of patients is usually small, so cost effectiveness should not generally be an issue when considering whether to use computed tomography, magnetic resonance imaging, or both [279]. A well structured, prospective study is needed to evaluate the efficacy of magnetic resonance imaging versus computed tomography for preoperative planning of histologically proven inverted nasal papilloma [279].

 Several radiologic patterns could be seen, therefore it is difficult to categorize any as specific for inverted papilloma [63, 280, 281]. A common radiologic presentation was a unilateral mass in the nasal fossa with opacification of the contiguous maxillary sinus in a moderately advanced tumor stage [63, 96]. The information required includes the presence and extent of disease involvement of the orbital wall, skull base, dura mater, and intracranial contents and of the great vessels. The presence of regional or distant metastases may also be determined. The real size of the tumor could be smaller than that shown in the radiological study [282, 283]. In the differential diagnosis, antrochoanal polyp, malignant sinus tumours and chronic rhinosinusitis, and fungal disease need to be excluded. The combination of bone deformity and sclerosis with the typical antromeatal mass suggests a slow-growing tumour such as IP [284].

#### 9.3.1. Computed Tomography (CT)

CT scanning is the ideal screening examination when a sinonasal tumor is suspected and the initial investigation of choice for assessment of a confirmed neoplasm [45, 79, 285]. The literature emphasizes the use of computed tomography scanning in management planning; higher resolution allows preoperative determination of the extent of disease, enabling the surgeon to plan the surgery more precisely [274, 285]. Scans should be obtained in both axial and coronal planes. The CT appearance of inverted papilloma is variable and nonspecific [276, 286–290]. Unilateral opacification of the paranasal sinuses is typical CT findings papilloma [99]. It could be difficult to differentiate from obstructed secretions or nasal polyps. Nonetheless, inverted papilloma is the most likely diagnosis when a unilateral mass in the nasal vault, producing benign bony changes, extends centrifugally into the maxillary and ethmoidal sinuses and through the nasal choana into the nasopharynx in an elderly patient with chronic nasal obstruction [284, 287]. Unilateral mass within the nasal cavity or paranasal sinuses with a lobulated surface configuration that appears on CT suggests IP as a primary diagnosis and also suggests IP in patients with tumor recurrence [284, 291]. Bony architecture is best shown on CT, with destruction and erosion of bone clearly shown in aggressive cases [45]. Bony changes on CT imaging of inverted papilloma (IP) are useful for predicting tumor origin and recurrence sites. Focal hyperostosis, bony strut, or osteitis detected on preoperative CT can predict with high degree of accuracy the site of origin [292–295]. The preoperative determination of tumor origin is important in selecting of the most appropriate surgical procedure [296]. Erosion, remodeling, and widening of the natural orifice of the sinuses on a CT scan are useful signs indicating IP. Calcification is usually, but not invariably, demonstrated in more benign processes and may calcify in punctate, nodular, linear, or circular patterns [6, 297]. Evidence of necrosis and hemorrhage may be seen, although this offers no definite diagnostic or prognostic information. Soft tissue invasion can also be demonstrated. Tumors have a soft tissue density which, if hypervascular, markedly enhances with the addition of intravenous contrast. Tumors can usually be distinguished from entrapped secretions, which have a lower density (that of water), although they may become isodense with tumor as they desiccate.

Although using multiple CT diagnostic signs provides a reasonable assessment of tumor origin and extent, accurate tumor mapping was still impossible because of inadequate differentiation of tumor from inflammatory pathologies [298]. This drawback may be overcome by a complementary MRI scan. Inflamed mucosa also has a soft tissue density which enhances with contrast and may potentially lead to overestimation of the size of some lesions. Since preoperative CT staging is inaccurate in 20% of cases, surgical planning should be flexible to provide for the need of the intraoperative findings [299]. It may also be possible to identify regional metastases if the CT scan includes the neck. Nodes are suspicious if they are greater than 15 mm in size, if they enhance peripherally, show central necrosis, or if their margins are ill defined [300]. In particular, the retropharyngeal nodes, which represent the first echelon nodes draining the posterior nasal cavity and sinuses, should be evaluated by radiological studies.

#### 9.3.2. Magnetic Resonance Imaging (MRI)

Not only MRI is especially good for detecting soft tissue changes, but also MRI is superior to CT scanning in distinguishing papillomas from inflammation. The main utility of MR imaging is in defining the extent of the lesion, providing better delineation of the lesions in contrast to surrounding soft tissue [284, 301, 302]. In most cases, MRI assessment of inverted papilloma can accurately predict the extent of tumor involvement. Iimura and colleagues [303] were able to demonstrate a high rate of agreement between diagnostic imaging and the actual surgical findings in identification of the origin of inverted papillomas. With gadolinium enhancement, MRI demonstrates perineural invasion and dural or intracranial involvement very well [13]. Since MRI provides excellent delineation of tumor from surrounding inflammatory soft tissue and retained secretions, preoperative staging of inverted papilloma by MRI is useful for planning an appropriate surgical approach [302, 304], and for selecting cases that can be managed by endoscopic approaches, resulting in lower rates of tumor recurrence and morbidity [305, 306]. Dynamic MR imaging can differentiate accurately recurrent IP from postoperative changes [307]. Lymph nodes can also be assessed with MRI with similar size and peripheral characteristics as for CT. Involved nodes are heterogeneous on T2 weighting and enhance peripherally on contrast scans using fat suppression [300, 308]. Further advantages of MRI are that images can be obtained in multiple planes, there is no exposure to ionizing radiation, and there is no artefact in the presence of dental fillings.

Ojiri et al. [309] discussed the following MRI features as potentially distinctive of sinonasal inverted papilloma on MR imaging. First, IPs have a heterogeneous appearance on MRI. On T1-weighted images, sinonasal papillomas appear slightly hyperintense to muscle; however, on T2-weighted images, SPs have intermediate signal intensity. A convoluted cerebriform pattern on T2 and enhanced T1-weighted MRIs for inverting papilloma may be potentially distinctive in 80% of cases [309]. Inflammatory polyps and inspissated material in the sinuses secondary to obstruction by the papilloma are hyperintense on T2-weighted images [304]. Although a convoluted cerebriform (CC) is a reliable MR imaging feature of sinonasal IPs, it can also be seen in various malignant sinonasal tumors. A focal loss of CC might be a clue to the diagnosis of IPs concomitant with malignancy [310]. A columnar pattern is a reliable MRI indicator of IP and reflects its histological architecture (positive predictive value of 95.8%). The combination of this finding with the absence of extended bone erosion allows for the confident discrimination of IPs from malignant tumors [276]; however MRI is unable to distinguish malignant and benign regions in a central mass with no gross necrosis, since this lesion does not alter the basic morphological pattern of inverted papilloma [311]. Because of the findings listed above, preoperative staging of IP by MRI can more accurately define the true extent of the lesion [304]. MRI was also recommended for postoperative followup [304].

#### 9.3.3. Positron Emission Tomography (PET)

Experience is growing with positron emission tomography (PET) using such agents as 18-F fluorodeoxyglucose and C-ll methionine to image the metabolic activity of a range of head and neck tumors, including those of the nasal cavity and paranasal sinuses. However, their routine clinical application is still under investigation [13]. The SUVmax value of a sinonasal tumor can warn the surgeon of the probability of an associated malignancy, even when preoperative biopsy demonstrates a purely benign papilloma [312]. F-FDG PET/CT demonstrating avid FDG uptake does not necessarily imply the presence of coexistent malignancy. Although IPs containing foci of SCC had consistently higher SUVs than IPs without SCC, the limited literature on this subject suggests that PET cannot be used reliably to make the distinction [313]. PET has the ability to distinguish between viable tumor and radiation—or surgery—induced fibrotic changes, which would make it a very useful form of followup imaging. For treatment planning, the anatomical detail provided by such studies remains inferior to CT and MRI [13, 313–315].

## 10. Management

The combined use of imaging techniques and diagnostic nasal endoscopy allows for an accurate diagnosis and enables minimally invasive techniques to be tailored to the patient's disease [316]. Intraoperative endoscopic examination is comparable in sensitivity but better in specificity than preoperative CT analysis for differentiating between IP and other diseases [283]. Early diagnosis of IP is essential for optimum management. Hence, the clinician should have a low threshold for suspecting the disease [317, 318]. Sisson et al. [319] appropriately recommended that any sinonasal complaint lasting 6 weeks or more should be assessed with a thorough history and head and neck examination, including assessment of cranial nerve function and an endoscopic evaluation of the nasal cavity and sinuses. A detailed history and physical examination should include close attention to the signs and symptoms of orbital extension and cranial nerve involvement. Any abnormal tissue should be biopsied and any suspicious mass assessed with CT scans. Those without suspicious findings are treated with a 2-week course of antibiotics and steroids and CT scans are performed on any whose symptoms do not resolve. By using this regime, he claimed a reduction from 8 months to 4 months in the average length of time from the onset of symptoms until a diagnosis of tumor [13]. Although CT and MRI can distinguish between what is tumor and what is not, in most instances the correct pathological diagnosis cannot be determined from radiological appearances. A tissue biopsy should be obtained prior to embarking on a plan of treatment, both to confirm the diagnosis and to ensure that the treatment proposed is appropriate [13]. In equivocal cases, increased proliferative activity as demonstrated by PCNA or Ki67 immunostaining may be useful in excluding a sinonasal polyp [209].

Absence of neurological symptoms at the time of diagnosis and first treatment of the disease does not eliminate a later on intracranial extension. Consequently, radical surgery and proper followup might prevent this unusual complication [57]. Selection of therapy depends on an accurate radiographic assessment of the extent of tumor. The tumor's local aggressiveness, high rate of recurrence, associated malignancy, and multicentric tendency have led most workers to advocate radical surgical removal of the tumor by lateral rhinotomy and en bloc resection of the ethmoid labyrinth. Radiographic evaluation by CT and MRI permits identification of a small group of patients who have limited lesions and may be candidates for conservative tumor resection by intranasal or transantral sphenoethmoidectomy [210].

## 11. Staging

Staging allows the assessment of outcome following different approaches. Also, having standardized grouping of IP is necessary to allow valid studies or meta-analysis [320]. Different from the TNM staging system of the American Joint Committee on Cancer (AJCC) and the International Union Against Cancer (UICC) for sinonasal tumors [321], IP mandated the development of its own staging system that suits its own characteristics [322]. Skolnik et al., 1966 [323], and Schneider, 1976 [322], developed a four-stage (T1–T4) staging system for IP. The higher the stage, the more advanced the disease. In T1 the tumor is confined to the nose, T2 or T3 are intermediate levels with variable involvement of the paranasal sinuses, and in T4 the disease has gone outside the nose or paranasal sinuses.

Krouse [325] introduced his staging system (Table 5) for inverted papilloma in 2000, for the assessment of resectability of the disease, with consideration of the difficulties encountered during the operation. Stage I disease is limited to the nasal cavity alone. Stage II disease is limited to the ethmoid sinuses and medial and superior portions of the maxillary sinuses. Stage III disease involves the lateral or inferior aspects of the maxillary sinuses or extension into the frontal or sphenoid sinuses. In addition to the Krouse staging system, T3 was further categorized by Oikawa et al. [305] as subgroup T3-B if tumors extended into the frontal sinus or the supraorbital recess; otherwise, they were categorized as T3-A. Stage IV disease involves tumor spread outside the confines of the nose and sinuses, as well as any malignancy. This important difference in location that addresses specific difficulties of endoscopic surgery was not considered by most of the existing staging systems. Krouse staging system for inverted papillomas can facilitate both treatment planning and comparison of surgical outcomes [326]. The categorization of IP on the basis of tumor origin enables a better surgical design and more accurate excision of the tumor. Although in some cases medially originated inverted papilloma (MOIP) may behave more aggressively, radical procedures were indicated in only the late Krouse stage laterally originated inverted papilloma (LOIP) without compromising the recurrence rate [327].

In 2007, Cannady et al. [328] developed a new staging system for IP providing information about prognosis for IP managed by advanced endoscopic techniques. This system categorized patients into three groups on the basis of recurrence rates (RR) (see Table 6). Cannady's classification provided objective data for preoperative planning and patient counseling in contrast to other staging systems that reflected surgeon's judgment rather than outcomes data [328]. The Krouse and Cannady systems provided a good distribution of patients according to local control [329].

Regional nodal metastases are uncommon [330]. Lymph node involvement is usually indicative of IP tumors associated with malignancy and extending locally to adjacent sites. The retropharyngeal nodes comprise the first echelon lymphatic drainage for sinus malignancies. Cervical lymph node are involved with T2 rather than T3 or T4 tumors of squamous cell histology [241, 308]. Other regional nodes that may be involved with lymphatic spread are the periparotid and level 1b nodes [257]. Distant metastases are also uncommon and could be seen with IP associated with malignancy or malignant transformation [331]. 

## 12. Treatment

The treatment algorithm for this tumor has undergone a complex evolution that continues today [234]. The rare occurrence of IP has limited the development and testing of treatment strategies. The majority of published data consists of retrospective reports from single institutions, which have inherent selection and treatment bias, comprise various histologies, and often combine all paranasal sinus and nasal cavity tumor sites. Treatments may include medical intervention, radiation therapy, surgical intervention, or a combination thereof. Early detection and aggressive local treatment are desirable [332].

As with other types of skull base tumors, a multimodality interdisciplinary team approach should be considered, with a tumor board consultation being recommended, including otorhinolaryngologist, head and neck surgeon and a neurosurgeon, and when indicated a neuroradiologist, pathologist, radiation oncologist, oncologist/neurooncologist, radiation oncologist, neurologist, neurophysiologist, ophthalmologist/neuroophthalmologist, oral/maxillofacial surgeon, and plastic surgeon as active members. Neurosurgery is needed for skull base involvement, intracranial extension, and potential dural involvement. Although multiple surgical and adjuvant approaches are available, a balance should be found in attempting to preserve cosmetic, oral, and nasal function. When possible, the orbit should be preserved and reconstructed. Visual acuity, evaluation of any extraocular motility disturbances, and proptosis should be documented through an ophthalmology consult. Postoperatively, the ophthalmologist may assist with treatment of epiphora or dry eye syndrome, with dental consultation to evaluate for dental extraction in preparation for radiotherapy. Consult a prosthodontist if maxillectomy is expected, and consult speech pathology as needed. Despite these seemingly competing needs, first and foremost should be the goal of a safe and complete eradication of disease, when possible [333].

### 12.1. Medical Therapy

The role of medical therapy is limited; it is used mainly as an adjunct to specific complications such as sinusitis. With some of the data suggesting the human papilloma virus may be a potential etiologic factor in the development of inverted papilloma, the use of antiviral agents may be beneficial. *Interferon* and other antiviral agents like intralesional therapy with Cidofovir has been suggested for the use of a patient with multiple recurrences, advanced disease, or spread to the orbit and skull base. This is still investigational [334, 335].

*Mitomycin C* was tried as an adjuvant therapy for dacryocystorhinostomy (DCR) approach in the treatment of Schneiderian papilloma of the nasolacrimal tract, and it showed preservation of function, compared with the conventional treatment of dacryocystectomy alone [336].

Preoperative medical therapy can help normalize inflamed mucosa and minimize intraoperative bleeding [337]. Adjunct chemotherapy can be used in malignancy associated IP [338]. A better understanding of alterations in epithelial cell proliferation and cell cycle regulation in inverted papilloma may lead to adjuvant medical therapies to decrease recurrence rates and improve treatment [339].

### 12.2. Radiotherapy

Radiation therapy is generally not a recommended option to surgery in the treatment of benign IP. Although surgery is generally the primary treatment modality for this disease, radiation therapy should be considered for patients with early or multiple recurrence, advanced stage lesions, biologically aggressive tumors [20], incompletely resected lesions [103] and lesions with positive margins, unresectable lesions, and tumors associated with malignancy [233, 340–344] or patients who are poor surgical candidates in whom the morbidity of the radical surgery would be intolerable [345] (Table 7). Combined surgical and radiation therapy appears to be an effective adjunctive procedure [16]. Radiation therapy carries a potential risk of malignant transformation in an otherwise benign lesion. There has been some concern with the use of radiation therapy for the treatment of inverted papilloma without an associated malignancy. Studies by Weissler et al. [346] and Mendenhall et al. [233, 347] have shown no conversion to malignancy and excellent control rates. Radiotherapy doses range from 64.8 to 74.4 Gy, depending on the amount of residual disease. Twice daily fractionation at 1.2 Gy per fraction is used to reduce the risk of visual damage. Proton beam radiotherapy may be used to deliver the boost portion of the treatment to further reduce the dose to normal tissues and reduce the risk of late complications [233].

### 12.3. Surgical Procedures

The primary and preferred treatment of inverted papilloma is surgery. Advances in the surgical approach to the anterior skull base have resulted in increased opportunity for the cure of tumors involving these structures. Traditionally, surgical resection has been the primary treatment modality for the management of sinonasal tumors involving the maxillary or ethmoid sinuses that are centrally located in the nose and sinuses [45, 348]; however, resection is often limited by critical structures such as eyes, brain, and the cranial nerves. A perfect surgical approach to nasal cavity and paranasal sinus tumors should provide a broad exposition preserving the important structures with no cosmetic defect [349]. Surgical advances are allowing more patients to enjoy functional reconstructions and better quality of life [350–352]. Surgery offers the optimum modality of treatment for inverted papilloma although a considerable range of operative approaches have been described. 

Precise determination of the sites of tumor origin and attachment during the operation, strict application of selection criteria, proper preoperative evaluations, intraoperative determination of extent and attachment of the tumor, close endoscopic followup, expert application of endoscopic techniques, meticulous use of subperiosteal dissection in the involved areas, wide removal of the tumour origin along the subperiosteal plane as well as drilling the underlying bone [353], complete removal of all diseased mucosa [2, 354] with creation of wide cavities, and long-term regular followup evaluation are the key elements to the successful treatment [355–364]. The extent of disease primarily determines the choice of surgical approach, with previous treatment, individual patient factors, and surgical expertise are secondary determinants [317, 365–369]. Limited involvement of the skull base can be successfully achieved by endoscopic excision [370]. Endoscopic resection of IP was associated with shorter hospital stay [371, 372], equivalent estimated blood loss [372], equivalent to shorter operative time [339, 371, 372], and lesser morbidity [339, 371]. The endoscopic approach should be performed by experienced surgeons and restricted to carefully selected patients with nasal, ethmoidal, and limited maxillary disease, if complete exposure of the tumor is possible, long-term followup is feasible, and there is no evidence of associated malignancy [373–376]. The intraoperative findings, most importantly the site(s) of tumor attachment, dictate whether an endoscopic procedure is sufficient to completely resecting the inverted papilloma or whether an adjunctive external procedure is required [377]. Although the endoscopic approach is gaining popularity for the treatment of inverted papilloma, indiscriminate application may result in a high recurrence rate. While one systematic review of the literature supports endoscopic resection as a favorable treatment option for most cases of sinonasal inverted papilloma; evidence based medicine (EBM) rating: B-3a [378] another systematic review reached the conclusion that there is not enough evidence in the literature to support endoscopic or open technique options for management of inverted papilloma [356].

Traditional surgery is reserved for more extensive lesions, recurrent lesions, or patients who have developed squamous cell carcinoma arising from an inverted papilloma [379–381]. Massive skull base erosion, intradural or intraorbital extension, brain invasion, extensive involvement of the frontal [382] sinus or infratemporal fossa involvement [383], abundant scar tissue caused by previous surgery determined by preoperative CT or MRI, or the concomitant presence of squamous cell carcinoma were considered limitations for a purely endoscopic approach [92, 230, 359, 384, 385]. With more extensive tumors and lesions in difficult locations, better visualization can be obtained by a combined limited external and endoscopic endonasal approach or by radical external approaches. IPs that arise from the lateral or inferior maxillary sinus are more difficult to remove endoscopically and are more likely to recur than IPs, which are sessile on medial parts of the maxillary sinus [386]. Hence Waitz and Wigand (1992) recommended the external approach for those IPs growing from peripheral regions of the sinuses [387, 388]. For treating I-II-IIIa stage nasal inverted papilloma, the nasal endoscopy endoscopic surgical technique was proved to be a better method [389]. Better results for patients in stage IIIb would be achieved by combining endoscopic technique with external approach in carefully selected patients [390, 391]. As to patients with stage IV, radical external approaches should be considered [392–396].

Early and aggressive surgery is usually recommended [60, 79, 217, 397–401]. Patients with inverted papilloma should undergo thorough surgery to remove the whole mucosal disease, most probably by the endoscopic, endonasal route when complete resection is possible [386, 402–404]. The whole tissue should be inspected for possible malignancy [90, 405]. The pathological examination of surgical margin could partially judge the prognosis [406]. Controversy exists as to whether the bony undersurface of an IP should be removed [95]. Partial and combined excision of the orbital plate of the ethmoid and lacrimal bones (extended operation of the extranasal ethmoid and frontal sinuses) has been recommended in cases in which tumors adhered to the orbital plate [407]. Cases demonstrating atypia or dysplasia may be treated by the endoscopic route [2]. The operating microscope is an extremely valuable tool for the surgical treatment of this lesion in light of the absolute necessity for its complete removal [408]. The application of shaver system was recommended [392]. The microdebrider may be used without altering the histopathology necessary for diagnosis [409, 410]. In order to decrease bleeding during the operation, the body and bases of the IP were irradiated with YAG laser [392]. Laser endoscopic coagulation seems to be an effective treatment for multicentric sinonasal inverted papilloma especially for lesions with a difficult surgical access [411]. Tumor excision with the 532 nm potassium titanyl phosphate (KTP-532) laser provided bloodless dissection and therefore excellent visualization [412]. Low-temperature plasma radiofrequency is a minimally invasive treatment for nasal inverted papilloma. Endoscopic surgery using the low-temperature plasma radiofrequency had the following advantages: less blood loss, complete en bloc tumour resection, mild injury, short surgery time, and mild postoperative pain [413, 414].

Growth in experience with the endoscopic management of inverted papillomas has led to evolving technical improvements and innovations. The recent literature has further refined the technique of endoscopic resection of inverted papillomas by delineating essential principles and applying new technologies, such as image guidance and angled endoscopic drills. Modified approaches and methodology have been described to address tumors originating in particular anatomic locations. In most tumor locations, the completeness of resection achieved by a skilled endoscopic surgeon [415, 416] is equivalent, if not superior, to that attained with an open approach. In some instances, an endoscopic exploration with tumor resection may help define the site of tumor attachment and direct adjunctive open procedures when indicated. Long-term outcomes studies with sizeable patient cohorts will be needed to define the role of various surgical strategies in the optimal management of inverted papilloma [417].

#### 12.3.1. Image Guided Surgery

A treatment based on endoscopic resection with image guidance appears to offer a safe treatment modality of inverted papilloma with insignificant morbidity [418]. The image-guidance system could identify the operative borders and critical anatomical structures together with the corresponding CT data, especially in cases with anatomical variations, unidentified anatomical landmarks, and intranasal or anterior skull base tumors. Endoscopic sinus surgery, combined with the image-guidance system, can provide accurate tumor resection while preserving normal tissue. It could increase surgical effectiveness and decrease overall surgical complications [419–422]. The improvement in surgical techniques, like the four-handed technique, has rendered endoscopic procedures capable of managing complex pathologies, according to the same surgical principles of the open approaches. From now onwards, frameless neuronavigation, modular approaches, intraoperative imaging systems, and robotic surgery are and will be an increasingly important part of endonasal surgery, and they will be overtaken by further evolution [423]. Intraoperative computer-aided surgery using magnetic resonance imaging (MRI)/computed tomography (CT) fusion imaging for (IP) could be helpful to surgeons when shifting fusion imaging when opening the frontal sinus bone to obtain CT data, and shifting to MRI to detect the tumor pedicle, then approaching the bone defect using MRI-CT fusion imaging (50-50%) [424].

#### 12.3.2. External Approach

External approach has been the golden standard for sinonasal tumor [425] removal but is associated with several side effects, including facial scars, intracranial and extracranial complications, a long hospitalization period, and high costs [348]. The external surgical approach is adequate for tumors extending to the brain, orbit, and maxillary sinus. Improved survival rates since the 1960s in all base-of-skull malignant tumors except for nasal carcinoma can be attributed to advances in surgical techniques [128]. External surgical approaches ranging from Caldwell-Luc to medial maxillectomy were introduced to reduce high local recurrence rates seen with traditional debulking procedures. However, reports show that acceptable control rates of about 85 percent can be achieved with less invasive endonasal endoscopic resections [378, 426]. Predominantly cases with nonmedical involvement of the maxillary sinus still require a supplement with the Caldwell Luc procedure [418]. Caldwell and Luc described the Caldwell-Luc operation more than 100 years ago as the surgical treatment for maxillary sinus disease. During the last decades less radical interventions using endoscopic approach have mainly replaced this classical procedures by Caldwell-Luc approach in endoscopic medial maxillectomy for treating inverted papilloma [427]. For disease extending to multiple sites, open surgical oncological procedures are associated with operative risk and postoperative morbidity and may not assure complete control of the tumor [428].

The available surgical techniques are as follows:

1. limited conservative surgery,
2. lateral rhinotomy,
3. transfacial with midface degloving,
4. craniofacial resection [429],
5. endoscopic approaches.

**Limited Conservative Operations**

Conservative surgery (e.g. anterior maxillary punch, polypectomy) is successful in controlling some cases, but may requires two or three operations. The surgical exposure in inverted papilloma should be adequate to ensure complete excision. Selected patients with limited size localized disease can be satisfactorily managed by conservative procedures if they are carefully followed [90, 430]. Limited procedure is considered justified even in cases with lateral lesions, if the tumor is sufficiently visible and confined. Septal IPs are often detected at an early stage and are therefore often amenable to local excision [217]. Even advanced IPs have small attachments. Their identification facilitates efficacious resection with minimal morbidity [431]. Because the lateral wall and floor of the sphenoid sinus are the most common origin and recurrence sites, the anterior wall of the sphenoid sinus should be opened as wide as the lateral wall and inferiorly to the level of the floor, especially in deeply pneumatized sphenoid sinuses [68]. A limited procedure is considered justified even in cases with lateral lesions, if the tumor is sufficiently visible and confined. Septal IPs are often detected at an early stage and are therefore often amenable to local excision [217, 281].

**Lateral Rhinotomy Approach and Medial Maxillectomy**

The traditional lateral rhinotomy incision described originally by Moure in 1902 has proved a versatile approach to the midfacial skeleton [432]. Most of the papers written in the past regarding surgical treatment of nasal and sinus inverted papilloma recommend aggressive surgical treatment—usually a medial maxillectomy with external or transantral ethmoidectomy. Lesions deep in the nasal vault with contiguous sinus involvement often required a lateral rhinotomy for exposure. This procedure affords excellent surgical access, but requires a significant external incision. The technique of lateral rhinotomy and en bloc excision of the lateral nasal wall, followed by meticulous removal of all mucosa in the ipsilateral paranasal sinuses, has been the standard therapy [285]. Most authors recommend lateral rhinotomy [65, 79, 231, 432, 433] as the initial surgical approach for all surgery on the midfacial skeleton and anterior skull base; however, conservative surgery has been reported effective in selected cases [90, 434]. Medial maxillectomy and ethmoidectomy via lateral rhinotomy reduced the recurrence rate dramatically [211, 435]. Technical refinements have allowed this procedure to be performed with acceptable and minimal morbidity. Its advantages are the excellent exposure provided, the opportunity to extend the approach to adjacent areas of tumor extension (orbit, cranial vault, frontal, and contralateral ethmoid sinus), and en bloc removal of neoplasms. This approach remains the standard of treatment for lateral nasal wall tumors [435]. Lateral rhinotomy performed in conjunction with operations not requiring inferior turbinectomy, such as anterior craniofacial resection, may adversely affect nasal airway function [436]. Lateral rhinotomy complication includes poor cosmesis due to depression of the nasofacial groove [432] but does not impart significant aesthetic morbidity [16, 90, 281, 437].

**Midfacial Degloving Approach**

Midfacial degloving is an extended sublabial rhinotomy, degloving constitutes a hidden route of access to the middle third of the face. It combines a bilateral sublabial incision with a rhinoplastic approach, whereby all the soft tissues of the face can be undermined subperiosteally, which permits good exposure of the midface, extended bilateral route of access to the nasal cavities and ethmoid/sphenoid/maxillary paranasal sinuses, nasopharynx, the base of the skull, and the clivus. It is an excellent alternative to the lateral rhinotomy in exposure to the nasal cavity, paranasal sinuses, and the nasopharynx. The exposure obtained using the degloving approach is superb. By lifting the soft tissue from the midportion of the face, many extensive benign inverted papilloma and some limited malignant lesions could be safely removed. This procedure may be associated with other neurosurgical approaches in the treatment of extended lesions of the base of the skull. Its use is particularly recommended in the treatment of inverted papillomas, especially in cases requiring bilateral surgery, although it applies as well to malignant tumors of the sinuses under certain conditions. It can be enlarged by supplementary incisions to meet the demands of tumour surgery [438–443]. This procedure is very versatile; it could be modified [444, 445] or combined with other approaches such as transtemporal, intracranial approach to resect more extensive tumors around the orbit, central skull base, as well as endoscopic approach [438–442, 446, 447]. It can also be extended to complete cervical-facial degloving for mandibular or mouth lesions, or if neck dissection is needed [443]. It provides excellent access to a wide range of resections, such as medial maxillectomy, radical maxillectomy with and without orbital exenteration, anterior skull base craniofacial resection, and partial rhinectomy.

The postoperative functional outcome is good with minimal complications and low recurrence [448]. Common complications related to midfacial degloving are temporary infraorbital anesthesia, hypoesthesia, temporary asymptomatic nasal vestibular stenosis, temporary nasal crusting and epistaxis, palatal dysfunction, oroantral fistula, rupture of subpetrous part of the internal carotid artery in one patient, and temporary facial palsy in another one [349, 443]. Radical sinus surgery with resection of the turbinates by means of midfacial degloving seems to disturb the climatization of the inspiratory air in the nasal cavity. Reduced absolute humidity and temperature may contribute to crusting, bleeding, and nasal dryness as frequent complaints of patients after aggressive sinus surgery with resection of the turbinates [449]. Because of absence of external skin incisions the cosmetic results are excellent with no visible external scars or facial deformity. It has proved to be an extensively valuable procedure for wide exposure of the operative [438–442, 450]. Lateral rhinotomy and midfacial degloving approaches have similar recurrence rates in T3 inverted papillomas but midfacial degloving has the advantage of no external facial scar and bilateral exposure [451].

Denker rhinotomy and medial maxillectomy through a sublabial technique, with dislocation of the nasal septum into the opposite nasal passage, provide excellent surgical exposure for these same lesions, while external facial incisions are avoided [452, 453]. A sublabial incision gives excellent exposure without external facial scar [247]. S. N. Wassef present a series of 23 patients treated over a ten-year period. Fifteen underwent an approach without an external incision. This recurrence rate lies between that reported for lateral rhinotomy and local transnasal excision [454].

**Craniofacial Resection**

IP of the paranasal sinuses can be removed thoroughly with a craniofacial approach that provides for good visualization. With this approach, some tumours previously considered inoperable may be removed and in some cases cured large extensive inverting papilloma of the ethmoid sinus that invaded the orbit and coursed over the globe around the optic nerve [429]. Skull base reconstruction may prove more difficult when there is significant frontal dead space resulting from chronic brain displacement by an associated mucocele [106]. Craniofacial resection and other aggressive treatments should be considered in IP with severe dysplasia for the associated high rate of recurrence and malignant transformation [455].

A modification technique of frontal craniotomy allows excellent access to the anterior cranial fossa and orbit. It is the approach of choice for orbital tumors. This technique results in en bloc removal of a bone flap which incorporates the orbital roof, superior orbital rim, and frontal bone. Its advantages are excellent exposure, clear visibility in the placement of chisel cuts, and less brain retraction than conventional techniques [456]. Endoscopically assisted anterior cranial skull base resection is an adjunct in the traditional anterior craniofacial resection [457]. The endoscopic-assisted craniofacial resection is a highly useful surgical technique to avoid the unsightly facial scar of the lateral rhinotomy or the Weber-Ferguson incision, postoperative paranasal sinuses infection, and tracheostomy in selected cases with a lower morbidity rate in selected cases [429, 458].

**Endoscopic Approach**

The advances in endonasal micro-endoscopic surgery allow both safe and effective removal of IP with low morbidity, and therefore it could be the approach of the first choice [459]. Functional endoscopic sinus surgery (FESS) is a video-assisted minimally invasive surgical procedure that opens up the sinus air cells and the sinus ostia with an endoscope. The use of FESS as a sinus surgical method has now become widely accepted and the term functional is meant to distinguish this type of endoscopic surgery from the nonendoscopic more conventional sinus procedure [460]. There has been an increasing popularity in the removal of nasal and paranasal sinus neoplasms through an endoscopic approach to minimize invasiveness. Endoscopic techniques have been used for treating increasingly complex intranasal pathology, including nasolacrimal duct obstruction, cerebrospinal fluid leaks/encephaloceles, dysthyroid orbitopathy, and optic neuropathy. The advent of rigid telescopes has revolutionized the management of skull base pathologies.

Endoscopic sinus surgery is a gold standard in the diagnosis and treatment of paranasal sinus inflammatory disease since 1970s [461–463]. More and more attention is put on endonasal tumor removal with the endoscopic technique [464, 465]. In the recent literature, emphasis has been toward the use of endoscopic surgical techniques in the management of benign sinonasal tumors especially IP, in contrast to the extensive open procedures recommended previously [230]. This trend has not been without controversy, given the association of inverted papillomas with malignancy [325]. The literature on endoscopic approaches to inverted papilloma consists primarily of relatively small case series (grade C evidence) [378]. Inverted papillomas of the sinuses, nasal cavity, and the skull base can be approached directly by using the endoscopic endonasal approach (EEA). This approach allows surgeons to see and access the tumor well without making incisions to the face or skull. EEA enables resection of benign and selected malignant sinonasal tumors and offers the benefits of no incisions to heal, no facial scars, no disfigurement to the patient, better functional and structural preservation of the sinonasal complex, minimal trauma to surrounding tissue, shorter recovery time, shorter hospitalization stay and lower costs, good success rates in preventing recurrence, excellent visualization, permiting removal of diseased mucosa while preserving vital anatomic structures, preserving the physiological properties of the mucosa while assuring proper ventilation of the nasal and sinus cavities [466], small bleeding, operation under magnification, good view around the corner in 70 degrees endoscope, leaving anterior bony wall of the maxillary sinus, leaving inferior turbinate and small postoperative disturbances, relatively small operative injury and quick healing, possibility of removing the tumor from the nose, ethmoidal and sphenoidal and maxillary sinuses (mostly), possibility to extend the operation with external approaches if needed, allowing for excellent postoperative surveillance, and better cosmetic outcomes than traditional open surgical approaches [348, 382, 428]. Endoscopic surgery proved to be successful even in large lesions affecting the posterior ethmoid, the sphenoid sinus, or the nasofrontal duct. After intranasal surgery patients with inverted papillomas have a better chance of retaining the bony framework with recovery of the respiratory and olfactory function [387]. The endoscopic approach is also recommended for special biopsies of tumors penetrating to the sphenoid sinus from the sella, the petrous apex, and adjacent areas. Endoscopic or endoscopic-assisted resection of IPs proves to be a valid technique with recurrence rates comparable with open approaches. Their survival results compared favourably to those reported with open surgery, and low morbidity and mortality rates, short hospital stays, and lack of facial incision were clear advantages [415, 426, 467]. Performing an endoscopic surgery of tumor one has to be prepared to make an intraoperative conversion to open surgery [464]. Concerns with EES include obtaining clear margins given anatomic constraints, tumor seeding from piecemeal resection, inaccurate preoperative prediction related to variable sizes, and locations of the IP bases, particularly in the maxillary sinus and the frontal recess [363, 382, 403, 468]. Resection of the inferior turbinate to perform an endoscopic medial maxillectomy can cause atrophic rhinitis and shrinking of the maxillary sinus, resulting in difficulty following these patients for recurrence in the office; this can be avoided by inferior turbinate preservation [469, 470].

#### 12.3.3. Tailored Approaches

The availability of different endoscopic techniques allows the entity of the dissection to be modulated in relation to the extent of disease [359]. Tailored endoscopic surgery is a safe and effective treatment that obviates the need for more extensive surgery for the management of inverted papilloma [360]. Endoscopic medial maxillectomy (EMM) is a safe, effective method for the treatment as well as for diagnosis and followup in all patients [283, 426, 471, 472]. Combined approaches can be employed, including adjuvant osteoplastic flap, midface degloving, trephine, or Caldwell-Luc approaches [273, 426]. The osteoplastic approach combined with endonasal surgery is suitable in far lateral located IP [459]. Inverted papilloma originating from the sphenoid sinus can be approached via endoscopic transethmoidal sphenoidotomy [473, 474]. Endoscopic sphenoidotomy is safe, with minimal blood loss and reduced operating time, allows maximal resection with minimal morbidity and short post-operative hospitalization, allows a direct route to the sphenoid sinus, and facilitates endoscopic postoperative surveillance [46, 47, 52, 54, 337, 475–478]. Extended endoscopic frontal sinusotomy may be necessary in the management of complicated frontal sinus inflammatory disease and inverting papilloma [479]. Rhino-frontal sinuseptotomy (RFS) is a combined intra- and extra-nasal approach reported for the surgical treatment of severely diseased frontal sinuses [480–482]. The limitations of endoscopic resection of inverted papilloma of the frontal recess can be managed with staged procedures, initial endoscopic resection of ethmoid/maxillary disease with subsequent open treatment of the frontal sinus [482]. Ethmoidectomy with wide antrostomy and sphenoidotomy can be used for IPs confined to the middle meatus. Medial maxillectomy with ethmoidectomy and sphenoidotomy can be used for IPs partially invading the maxillary sinus. Sturmann-Canfield operation is used for IPs involving the mucosa of the alveolar recess or of the anterolateral corner of the maxillary sinus [359]. The osteoplastic approach combined with endonasal surgery is suitable in far lateral located IP [459].

#### 12.3.4. Extended Endoscopic Approach

Cases with invasive disease or previous medial maxillectomy are treated via the open approach. Otherwise, the extended endoscopic approach is used [483]. The tumour is debulked, and its attachment points are identified. Endoscopic transnasal medial maxillectomy is then performed [484]. If maxillary sinus involvement in its anterior, inferior, superior, or lateral portion is suspected, a Caldwell-Luc approach is performed to allow for mucosal excision and complete removal of the anterior lateral nasal wall [485]. When the lamina papyracea or ceiling of the ethmoid or sphenoid sinus is involved, the bony wall is resected [407]. The frontal recess can be approached via a Lynch incision or an endoscopic transorbital approach. Extended endoscopic approach offers a safe, effective, and aesthetically acceptable [483, 486] reproducible technique with less operative time and morbidity, strong illumination, better magnification, superior resolution, angled visualization, coupled with exact osteotomies, and, possibly, better pathological tumor mapping than open medial maxillectomy for selected patients [487]. It is an endoscopic approach that is gaining acceptance as an alternative to craniofacial or transfacial tumor resection [481, 488, 489]. Partial and combined excision of the orbital plate is indispensable, in progressive inverted papilloma cases, to reduce recurrence [407]. Chemotherapy and radiotherapy are added, depending on the type of tumor and its extent [407]. In very carefully selected cases of malignant tumors ESS is oncologically acceptable, but more experience is needed for discerning the indications for endoscopic resection of malignant tumors [348, 490]. In most cases it is possible to achieve a good result through an endonasal approach. External approaches are recommended in T4 tumors or carcinomas. A longtime followup is recommended for each case [491]. Endoscopic removal of malignant lesions remains controversial because of the small number of patients and short postoperative observation [492]. Opponents to the endoscopic approach claim that endoscopic resection is carried out by piecemeal resection rather than by en bloc resection, the latter being a fundamental rule of oncologic surgery. En bloc resection is not necessary to achieve oncologic cure. However, several factors have to be considered before selection of this surgical approach. Large tumor size, intracranial or orbital extension, and extensive frontal or infratemporal fossa involvement are relative, but not absolute limitations [230]. In some cases, debulking of the tumor is needed to identify the origin of the lesion. Once this has been achieved the origin of the tumor can then be resected en bloc. Furthermore, meticulous use of frozen sections is of utmost importance for achieving an acceptable oncologic result [493].

## 13. Challenges and Complications

There are two main clinical problems that require particular attention in the management of skull base IP: the tendency for recurrence, and the risk of malignancy [16, 210]. Recurrent disease and metachronous carcinoma can develop after a prolonged period of time. Long-term followup is recommended to detect recurrence, as disease can become quite extensive before it becomes symptomatic [2]. The detection and definition of factors that allow a prognosis of recurrence or malignant transformation of inverted papilloma are an active field of research [494]. There is a considerable variation in frequency and incidence of reported complications, which possibly could be related to different followup periods and cohort sizes [495]. There are many reports of a change from primarily benign IP to carcinoma *in situ* and to invasive carcinoma [496, 497].

### 13.1. Recurrence

The magnitude of the recurrences is directly proportional to the completeness of removal with the best results obtained by techniques that afford the best operative exposure [23]. The incidence of recurrence tends to show variable and high rates; about 6–75% of cases will have recurrence after surgical removal [21, 60, 79, 211, 231, 246, 282, 401, 426, 433, 448, 468, 491, 498–506]. The average disease-free period ranges from 6 months to more [66, 219, 232] with most recurrences occurring at an average of 24−40 months after the procedure [426, 507, 508]. Multiple recurrences (without malignancy) were related to young age [509], smoking [108, 509], a mitotic index greater than or equal to 2 per HPF, an absence of inflammatory polyps, frontal sinus origin, increase in hyperkeratosis, the presence of squamous epithelial hyperplasia, increase in the mitotic index [89], epithelial atypia, and extent of lesions [397, 510, 511]. There was a correlation between the DNA ploidy and recurrence rate of IP; aneuploid tumors have a higher recurrence rate than diploid and multieuploid tumors. Quantitative measurements of DNA contents and morphological parameters may be important for early detection of recurrence and malignant change in IP [169, 512]. Recurrence was also related to HPV infection, which may be attributed to the wider range of infected cells in these cases [144]. Females showed higher recurrence rates than males [397]. Technological advancements have led to a trend of detecting sinonasal inverting papilloma before extension beyond the sinonasal region. While some authors referred most recurrences to incomplete resection [354], other authors denied that excision with safe margins, tumor extension, associated malignancy, or dysplasia have any significant impact on disease-free survival regardless of surgical procedure [379]. No significant differences were observed between the recurrence rates following endoscopic procedures, simple excision, open approaches, or combined resection [344, 426, 510, 513], but recurrence rates were higher with limited and conservative resections such as nasal polypectomy and Caldwell-Luc approaches [2, 218, 232, 235, 320, 324, 401, 425, 433, 506, 514]. Other studies showed a higher rate of recurrence among groups of patient treated with endoscopic resection compared to traditional surgical approach [355, 372, 515–517]. Results of conservative surgery in selected cases were comparable to those using radical methods [227, 505]. The estimated malignant potential for recurrent disease was up to 11 percent [2]. In cases with malignancies, the recurrence rate after the original surgical procedure was 22 percent [401]. Patients with locoregionally recurrent disease are treated with multimodality therapy, including resection, chemotherapy, and/or radiation therapy. IPs that recur after treatment may represent a subset of lesions with an inherent aggressiveness, for which optimal treatment has yet to be determined [505]. Recurrent inverted papilloma tends to behave more aggressively and has a higher postoperative recurrence rate than the primary lesion [358].

### 13.2. Malignancy

There is also a variable wide range in the frequency of reported carcinoma in patients with IP, ranging somewhere between 1–50% [1, 21, 79, 91, 210, 225, 231, 246, 281, 282, 285, 398, 491, 503, 504, 518, 519]. Carcinoma may occur *with* IP (synchronous carcinoma) or at a *later* time (metachronous carcinoma). Why carcinomas in inverted papillomas arise meta—or synchronous is also still unknown. The majority of cases in the literature are synchronous carcinomas [1, 2, 42, 217, 285, 354, 401, 518, 520]. Sinonasal carcinomas arise in about 10% of patients with inverted papillomas [1, 401]. The average transformation rate of metachronous carcinomas was reported about 2–7 percent with a mean time of 52 months (range: six to 180 months) [2, 225, 381, 400, 401, 505]. 

Malignancy was found to be associated with bilateral inverted papilloma, histologic multicentricity [521], a predominance of mature squamous epithelium, the presence of all three epithelial types (metaplastic squamous, mature squamous, and cylindrical), severe hyperkeratosis, a mitotic index greater than or equal to 2 per high-power field (HPF), absence of inflammatory polyps among the papillomas, an abundance of plasma cells, an absence of neutrophils, the presence of bone invasion, the absence of inflammatory polyp, increase in the ratio neoplastic epithelium : stroma, increase in hyperkeratosis, the presence of squamous epithelial hyperplasia, increase in the mitotic index, and a decrease in the number of eosinophils [511, 522]. Clinically benign behavior was related to the presence of inflammatory polyp and the absence of hyperkeratosis, predominantly mucinous tumors, a mitotic index less than 1 per HPF, a ratio of neoplastic epithelium/connective tissue stroma greater than or equal to 6, and the presence of inflammatory polyps among the papillomas [89, 511]. Precancerous lesions of IP exhibited elevated levels of EGFR and TGF-alpha and this expression may be associated with early events in IP carcinogenesis [139, 140, 174]. HPV infection may be an early event in a multistep process of malignant formation of IP [110, 139, 140, 143, 144, 164, 179, 523, 524]. Precancerous lesions of IP exhibit elevated levels of fascin that may be associated with carcinogenesis of IP [525, 526]. 

Patients with associated squamous cell carcinoma were found to be in older age groups, were more likely to manifest epistaxis than the more common unilateral nasal obstructive symptoms, and had less time between the onset of symptoms and presentation than those with inverted papillomas alone [527]. Male gender, advanced age, and recurrent inverted papilloma do not per se present risk factors for the development of associated malignancies [1]. Distant metastatic disease is typically treated with chemotherapy alone. 

Therefore, this emphasizes the importance of long-term followup with clinical examination and endoscopic. CT scan has been indicated for all patients with inverted papilloma to detect recurrences ad malignancy in all patients [225, 426]. The National Comprehensive Cancer Network (NCCN) guidelines recommend followup every 6 to 12 months after five years and suggest chest imaging as indicated [241, 528].

### 13.3. Complications

Complications of treating sinus malignancies are related to the extent of surgery [509] and reconstruction. Surgical complications include clinically significant bleeding, CSF leak, infection, anosmia, dysgeusia, and other cranial nerve damage. 

#### 13.3.1. Bleeding

Bleeding may occur if control of the large vessels is overlooked. This problem may occur if the artery is initially in vasospasm and if no active bleeding is noted until after surgery. The anterior and posterior ethmoid and sphenopalatine arteries may be cauterized, clipped, or ligated to prevent or control bleeding. If needed, interventional radiology may be requested to assist with intra-arterial coiling to control bleeding.

#### 13.3.2. CSF Leaks

Cerebrospinal fluid (CSF) rhinorrhoea and temporary nontension pneumocephalus are rare complications [344, 529–531]. During surgery, CSF leaks may occur near the skull base. Appropriate management starts with identification. Symptoms may include clear rhinorrhea, salty taste in the mouth, halo sign, or reservoir sign. Once noted, identification of the leak can be made endoscopically or with intrathecal injection of fluorescein. Tests, such as a test for tau or beta transferrin, may be most specific but may take days for results to be processed.

Conservative management with bed rest and a lumbar drain can be used for the first 5 days in addition to placement on antibiotics. If resolution has not occurred, surgical intervention should be used, including patching with a dermal allograft, turbinate bone, and nasal mucosa. Mucosal flaps can be elevated and used to close the leaks with interpositioned bone or cartilage. For large leaks, a spinal drain may be necessary to allow grafts and sealing techniques to solidify and integrate. Cerebrospinal fluid leaks can be successfully repaired endoscopically [426]. In patients with extensive pathology and difficulty of access, a more traditional lateral rhinotomy approach can result in a more successful outcome [529]. Reconstructive surgery to treat postoperative CSF leak using Surgicel, lyophilized dura, and TachoComb with Tissucol to close dural fistula was effective [531]. 

#### 13.3.3. Epiphora

Epiphora is a common complication of surgery caused by obstruction in the lacrimal outflow tract [344]. This can happen because of damage to the lacrimal puncta, sac, or duct. Care should be taken to marsupialize the lacrimal duct if it is lacerated or damaged in surgery to prevent outflow obstruction. Followup endoscopic or open dacryocystorhinostomy may be necessary [532]. The incidence of prolonged epiphora was not affected by postoperative radiotherapy, or timing of tube removal. Silicone tube stenting can be used as the effective and convenient transected nasolacrimal duct reconstructive technique to prevent prolonged epiphora [533].

#### 13.3.4. Diplopia

Diplopia is a known complication in any surgery involving the orbital cone. Proper repair of the orbital floor is a key to prevent this complication, but in some cases it is unavoidable even with meticulous reconstruction. In cases of diplopia, prism lenses are usually the simplest method for correction, as surgical correction by ophthalmology can be complicated by prior scarring from surgery and radiation treatment. Ophthalmology consultation is standard of care.

**Reconstruction**

Facial disfiguration is one of the most important patient concerns and can lead to considerable social and psychological stress. This outcome must be dealt with initially and on an ongoing basis. However, the ability to resect and reconstruct has improved significantly [423]. Without support, the esthetic results and airway patency may be compromised. Surgical or prosthetic reconstruction may be considered for large postsurgical defect. Surgical reconstruction of such a defect depends on support of the reconstructive tissues to prevent collapse [534]. The reverse buccinator musculomucosal flap is supplied by the retrograde blood flow of the anterior buccal artery and sutured to the defect through the oronasal tunnel. can be used for repairing the defect following nasal IP resection. The technique provides the solution to prevent nasal stricture from cicatricial contracture after excising inverted papilloma and can be performed simultaneously, saving another operation for the secondary deformity [535].

One of the most difficult decisions made during surgery is when to take or leave the orbit. Suarez reviewed the literature and had 3 recommendations [536]. First, close scrutiny of the periorbita is key when deciding for or against exenteration. Although the lamina papyracea and lacrimal bones can be invaded and destroyed quite quickly, the periorbitum is a much better barrier to invasion. So despite bony destruction, if the periorbita is considered intact, they make the argument for orbital preservation and reconstruction [407]. Once the periorbital has been violated, orbital exenteration is required because few barriers to spread exist within the orbital contents. Second, take into account the cancer histology. When dealing with more aggressive histology such as adenocarcinoma and SCC, a lower threshold for choosing exenterations would be expected, as opposed to other tumors with less local regional recurrence rates. Third, reconstruction is essential for large defects resulting from total orbital floor resection involving 2 or more orbital walls to prevent displacement and dysfunction of the eye [536].

If the eye is preserved, postoperative radiation is usually recommended. Nonetheless, patients should be counseled that despite surgical orbital preservation, impairment can occur from radiation, including optic atrophy, cataracts, dry eye, and ectropion. In the ideal cases, reconstruction preserves form and function. A free rectus flap or other distant tissue may be required to protect vital structures, or facial prosthetics may be used. Facial prosthesis can be offered to improve cosmetic results, but meticulous maintenance of the prosthesis by the team and patient is imperative.

## 14. Posttreatment Followup

Close endoscopic followup is mandatory to ensure early recognition and treatment of recurrent disease [484, 504, 537–539]. Propensity for delayed recurrences and incidence of malignant transformation mandates careful, long-term followup since the recurrence of tumor can happen after a long time [2, 139, 459, 513]. The disease can become quite extensive before it becomes symptomatic [2]. The recommended follow-up period is not clearly defined in the literature; it varies from at least two years to a lifelong followup [218, 426, 485, 540]. Lifelong followup is recommended for possible late recurrences and metachronous multifocal disease [507, 508]. It is suggested to do annual followup roentgenological examinations, preferably with the aid of computed tomography [96, 298]. Despite the lack of defined survival benefit from any posttreatment surveillance strategy in head and neck cancer, surveillance protocols are in widespread clinical use [541]. In general, the intensity of followup is greatest in the first two to three years, which is the period of greatest risk for disease recurrence [542–544].

Complete examinations are performed by trained surgeon at regular intervals independent of the mode of treatment or the stage of disease. The posttreatment periodic physical examination should be methodical and comprehensive. The anterior nasal cavity can be evaluated with a nasal speculum, but when necessary, complete evaluation of the posterior nasal cavity and nasopharynx requires fiberoptic endoscopy. Aside from mucosal irregularities, other abnormalities that should be specifically searched for are pooling of secretions, tissue asymmetries, or bleeding. Examination of the ears should note serous or acute otitis media which can indicate eustachian tube blockage by an obstructing nasopharyngeal mass. Parameters such as hyperkeratosis, squamous epithelial hyperplasia, and a high mitotic index are negative prognostic indicators, which could be useful in the future followup of patients with IP [139]. An additional followup modality that is becoming more routinely available in the head and neck surgeon's office is ultrasonography. Ultrasonography provides objective assessment of neck adenopathy and is especially helpful in the difficult to palpate posttreatment neck which often has “woody” induration [545].

## 15. Conclusion

There are no prospective long-term studies with sufficiently large cohorts of equivalently staged patients. Today, there is no single right or wrong method, but a range from which to choose in the individual case. The true population base for reported figures is uncertain, given that many series were reported from tertiary centres, where recurrent and problematic cases are likely to be overrepresented [2]. Because of the paucity of these skull base IP, a multi-institutional prospective collaborative study is needed [131]. 

Endoscopic nasal and sinus diagnosis and surgery are appropriate for diagnosis, followup, and treatment of both limited and recurrent inverted papilloma, provided all patients are made aware of the possibility of recurrence and need for more extensive surgery as a result [386]. Biopsies, CT and/or MRI must be performed for diagnosis and preoperative planning, and in any case with recurrence suspicion. Treatment for inverted papilloma is primarily surgical and increasingly using endoscopy [92]. The optimum surgical approach is determined by the extent of disease. Other factors in the choice of surgery are the surgical expertise, particularly in endoscopic IP surgery [417], previous treatment, and individual patient factors surgical approach choice must consider several parameters such as neoplasm localization, extension, dimension, and frontal recess anatomic features [317]. The stress is put on the need for radical excision under endoscopic control which can be combined for the cure of tumors impossible to be reached under endoscopic control only. Endoscopic resection of IP should be considered before the external approach is used [348]. Treatment of inverted papilloma with limited involvement of the skull base can be successfully achieved by endoscopic excision. Radiation is added if it is associated with squamous cell carcinoma or local recurrence, or if it is incompletely resected [546]. Irrespective of the tumor extent and of the approaches that was used for excision a close endoscopic followup still remain mandatory, by trained surgeons, in the long-term management of IP. Parameters such as hyperkeratosis, squamous epithelial hyperplasia and a high mitotic index are negative prognostic indicators, which could be useful in the future followup of patients with IP [139]. A better understanding of alterations in epithelial cell proliferation and cell cycle regulation in inverted papilloma may lead to adjuvant medical therapies to decrease recurrence rates and improve treatment.

## Figures and Tables

**Table 1:** Comparing IP to other Schneiderian papilloma.

Papilloma	Fungiform	Inverted	Oncocytic Schneiderian
Former name/synonyms	Septal	Ringertz	Cylindrical, columnar

Prevalence %	50	47	3–5

Origin	Nasal septum	Lateral nasal wall and paranasal sinuses	Lateral nasal wall and paranasal sinuses

Epithelium pattern of growth	Everted, exophytic	Infolded, endophytic	

Microscopy	Thick squamous epithelium and, less frequently, respiratory epithelium arranged in papillary fronds	Thickened squamous epithelium admixed with mucocytes and intraepithelial mucous cysts	Multilayered epithelium with an eosinophilic cytoplasm among which intraepithelial mucin cysts

Age group	Younger	50~60	30~80

Malignancy	35% have invasive squamous cell carcinoma	Locally aggressive, extending into the sinuses, the orbit, nasopharynx [20], or meninges. Three to 24% (mean 13%) may have an invasive focus of squamous cell carcinoma	14%~19% Malignant change potential

	25% multifocal		Mixed with typical inverted papilloma

**Table 2:** Presenting Symptoms.

Nasal cavity lesions	Sinus lesions
Earlier	Later
Obstruction	Obstructive symptoms
Bleeding	Postnasal drip
Pansinusitis	Congestion pain

**Table 3:** List of common symptoms.

List of symptoms
(i) Nasal obstruction, usually one sided
(ii) Rhinorrhea (runny nose) discharge
(iii) Epistaxis (nosebleed)
(iv) Sinusitis
(v) Facial pain
(vi) Loss of sense of smell
(vii) Frontal headache

**Table 4:** Signs and symptoms of invading IP, classified by site of invasion.

Intracranial Invasion	Symptoms	Signs
Anterior cranial fossa via the cribriform plate	Anosmia or symptoms related to displacement of the globe	Anosmia, proptosis

Orbital involvement	Proptosis, periorbital swelling, epiphora, diplopia, blurred vision, trigeminal paresthesias, trismus, retroorbital pain and visual loss, or complain of facial numbness	Impaired ocular mobility, anesthesia or hypesthesia of the infraorbital, sphenopalatine, or greater palatine nerves

Inferior extension into the oral cavity	Painful loose teeth, poorly fitting dentures, mass in the oral cavity, swelling of cheek, nose, or around the eye.	Oral cavity mass on the palate, upper Alveolus, or upper gingivobuccal sulcus or with malocclusion or loose teeth

**Table 5:** Krause staging system (2000) for inverted papilloma [324].

Stage	Location and spread	Malignant changes
T1	Tumor totally confined to the nasal cavity, without extension into the sinuses. (The tumor can be localized to one wall or region of the nasal cavity or can be bulky and extensive within the nasal cavity but must not extend into the sinuses or into extranasal compartment)	There must be no concurrent malignancy

T2	Tumor involving the ostiomeatal complex, and ethmoid sinuses, or the medial portion of the maxillary sinus, with or without involvement of the nasal cavity.	There must be no concurrent malignancy

T3	Tumor involving the lateral, inferior, superior, anterior, or posterior walls of the maxillary sinus, the sphenoid sinus, or the frontal sinus with or without involvement of the medial portion of the maxillary sinus, the ethmoid sinuses, or the nasal cavity	There must be no concurrent malignancy

T4	All tumors with any extranasal/extrasinus extension to involve adjacent, contiguous structures such as the orbit, the intracranial compartment, or the pterygomaxillary space	All tumors associated with malignancy

**Table 6:** Cannady's et al. staging system about prognosis (as operationally defined by RR) for IP managed by advanced endoscopic techniques.

Group	Location and spread	Recurrence rate
Group A	IP confined to the nasal cavity, ethmoid sinus, and medial maxillary sinus	3.0%
Group B	IP with lateral maxillary sinus, sphenoid sinus, or frontal sinus involvement	19.8%
Group C	IP with extrasinus extension	35.3%

**Table 7:** Indications of radiotherapy.

Indications of radiotherapy in IP
Patients unwilling or unable to undergo surgery
Poor surgical candidates
Intolerable morbidity of the radical surgery
Advanced and biologically aggressive SPs
Associated malignancy
Incompletely resected IP
Unresectable lesions
Early recurrence

## Abbreviations

CCP: Convoluted cerebriform papilloma
CT: Computed tomography
EBM: Evidence based medicine
EBV: Epstein-Barr virus
EEA: Endoscopic endonasal approach
EMM: Endoscopic medial maxillectomy
ESS: Endoscopic sinus surgery
EGF: Epidermal growth factor
EGFR: Epidermal growth factor receptor
HPV: Human papilloma virus
IP: Inverted papilloma
LOIP: Laterally originated inverted papilloma
MOIP: Medially originated inverted papilloma
MRI: Magnetic resonance imaging
NA-PNA: Neuraminidase pretreatment
PCNA-LI: Proliferating cell nuclear antigen labeling index
SCC: Squamous cell carcinoma
TGF: Tumor growth factor-alpha
WHO: World Health Organization.
